# Percutaneous Treatment of a Giant Coronary Artery Aneurysm Using a Covered Stent With a Drug-Eluting Stent “Sandwich” Technique: A Case Report

**DOI:** 10.7759/cureus.96320

**Published:** 2025-11-07

**Authors:** Aishwarya Umashankar, Montasir H Ali, Mostafa Abdelmaaboud, Adrian Ionescu

**Affiliations:** 1 General Medicine, Swansea Bay University Health Board, Swansea, GBR; 2 Cardiology, Swansea Bay University Health Board, Swansea, GBR

**Keywords:** acute coronary syndrome, cardiogenic chest pain, coronary aneurysm, covered stent, drug eluting stent, intravenous ultrasound (ivus), non-atherosclerosis, sandwich technique

## Abstract

Coronary artery aneurysms (CAAs) pose diagnostic and therapeutic challenges due to their rarity and potential for serious complications. We report a case of a middle-aged woman who presented with acute coronary syndrome and was found to have an isolated proximal right CAA. An anatomical assessment using intravenous ultrasound, followed by the application of the sandwich technique wherein a drug-eluting stent is used within the covered stent, was performed. This case highlights the importance of anatomical assessment, procedural planning, and evolving interventional techniques to manage non-atherosclerotic causes of acute coronary syndrome, like a CAA.

## Introduction

Coronary artery aneurysm (CAA) is an enlargement of the coronary artery lumen more than 1.5 times the diameter of an adjacent normal segment and is considered giant when it exceeds four times the reference diameter or measures >20mm [1,2]. It is a rare disorder found in up to 5% of coronary angiography, which remains the gold standard diagnostic test. The natural history of this condition remains unclear; however, the main cause for CAA in adults is atherosclerosis (90%), and in children is Kawasaki disease [3]. Non-atherosclerotic causes include connective tissue diseases, fibromuscular dysplasia, vasculitis, iatrogenic, idiopathic and autoimmune diseases [4]. These can be differentiated through various investigations. It carries a risk of complications: rupture, thrombus formation with distal embolisation and compression of adjacent structures. Although there are no guideline-specific recommendations in managing CAA, the percutaneous approach to exclude the aneurysmal sac by covered stents (CS) or coiling is widely adopted [5]. We describe a case of a 55-year-old woman who presented with acute coronary syndrome (ACS) and was subsequently found to have an isolated giant proximal right coronary artery (RCA) aneurysm. This case report focuses on anatomical assessment, some technical challenges and the stenting technique.

## Case presentation

A 55-year-old woman with hypercholesterolaemia and a history of smoking presented with chest pain. Her electrocardiogram (ECG) showed T-wave inversions in the inferior leads, and troponin level was mildly elevated (317ng/L; upper limit of normal <14ng/L). She was treated as a non-ST elevation ACS (NSTE-ACS) with dual antiplatelet therapy (DAPT), secondary prevention medications and subsequently listed for invasive coronary angiography.

**Figure 1:**
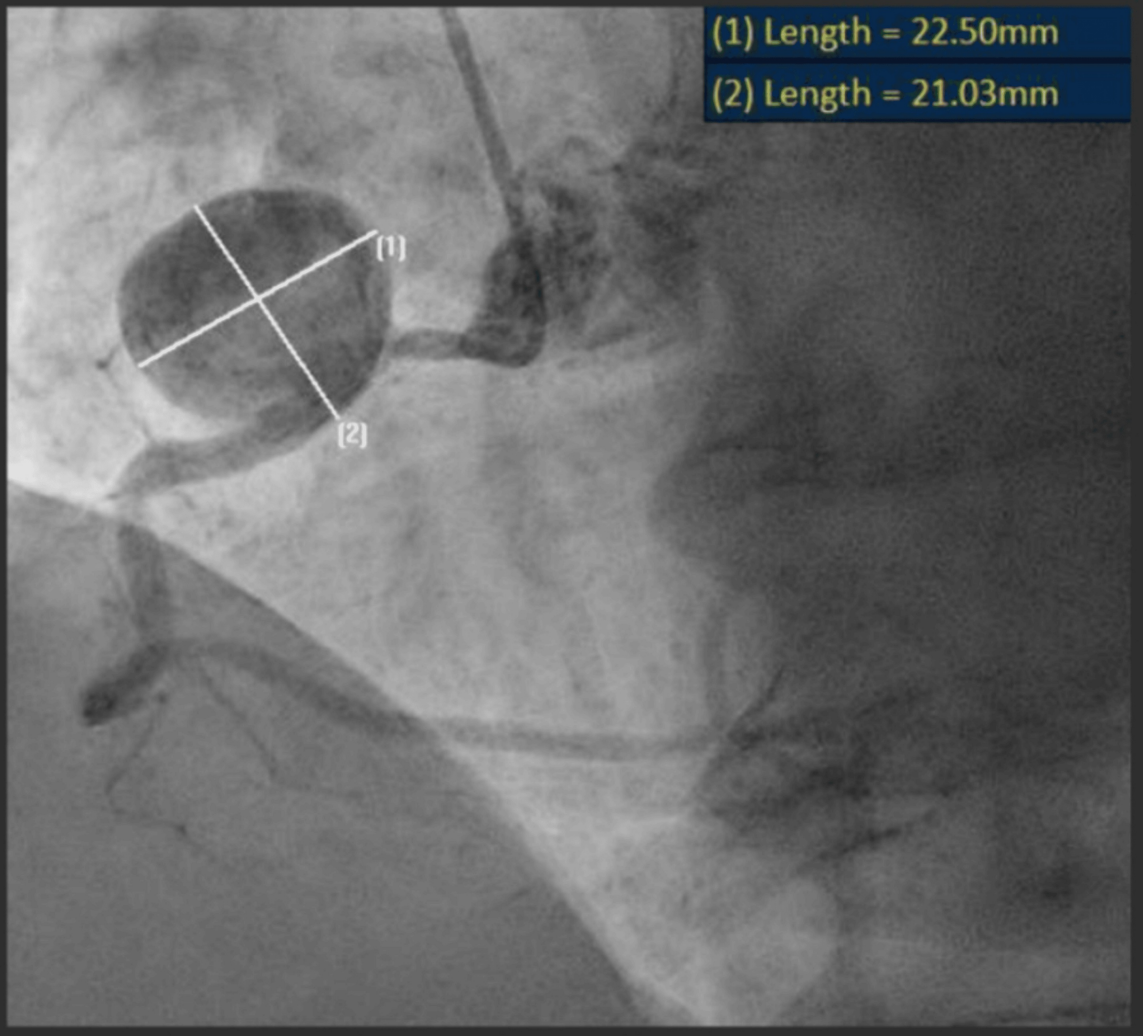
Right coronary artery aneurysm dimensions on angiography

**Figure 2:**
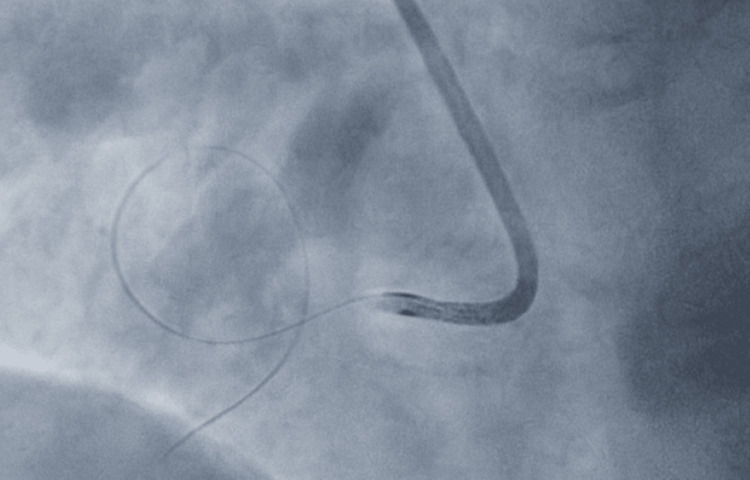
360-degree looping of the guidewire

Coronary angiography identified a giant, spherical aneurysm in the proximal RCA measuring approximately 22 x 21mm, as shown in Figure 1 and an aneurysmal mouth length of 0.34cm, as shown in Figure 2. The rest of the RCA and the left coronary artery had no aneurysms or flow-limiting disease. In the absence of any other alternative explanation, the large RCA aneurysm was thought to be the culprit of the patient’s presentation, likely due to in situ thrombosis followed by distal embolisation, or by reducing the RCA flow due to mechanical compression. After counselling the patient, a joint decision was made to intervene percutaneously.

A Judkins Right 4 guiding catheter (Cordis, Miami Lakes, FL, USA) and a SION Blue guide wire (Asahi Intecc, Japan) were used. The wiring was initially challenging due to recurrent looping inside the aneurysmal cavity despite trying different secondary curve shapes. This could be due to the large “mouth” of the aneurysm. Eventually, the guide wire was allowed to loop 360 degrees inside the aneurysm and exit towards the distal RCA as depicted in Figure 2.

A 2.5x15mm semi-compliant balloon navigated surprisingly smoothly over the guide wire and its loop inside the aneurysm, and was deployed distally as a distal anchor. Gentle traction on the fixed guide wire "unlooped" it, and it "fell" into the proximal RCA lumen, leaving the aneurysm.

**Figure 3:**
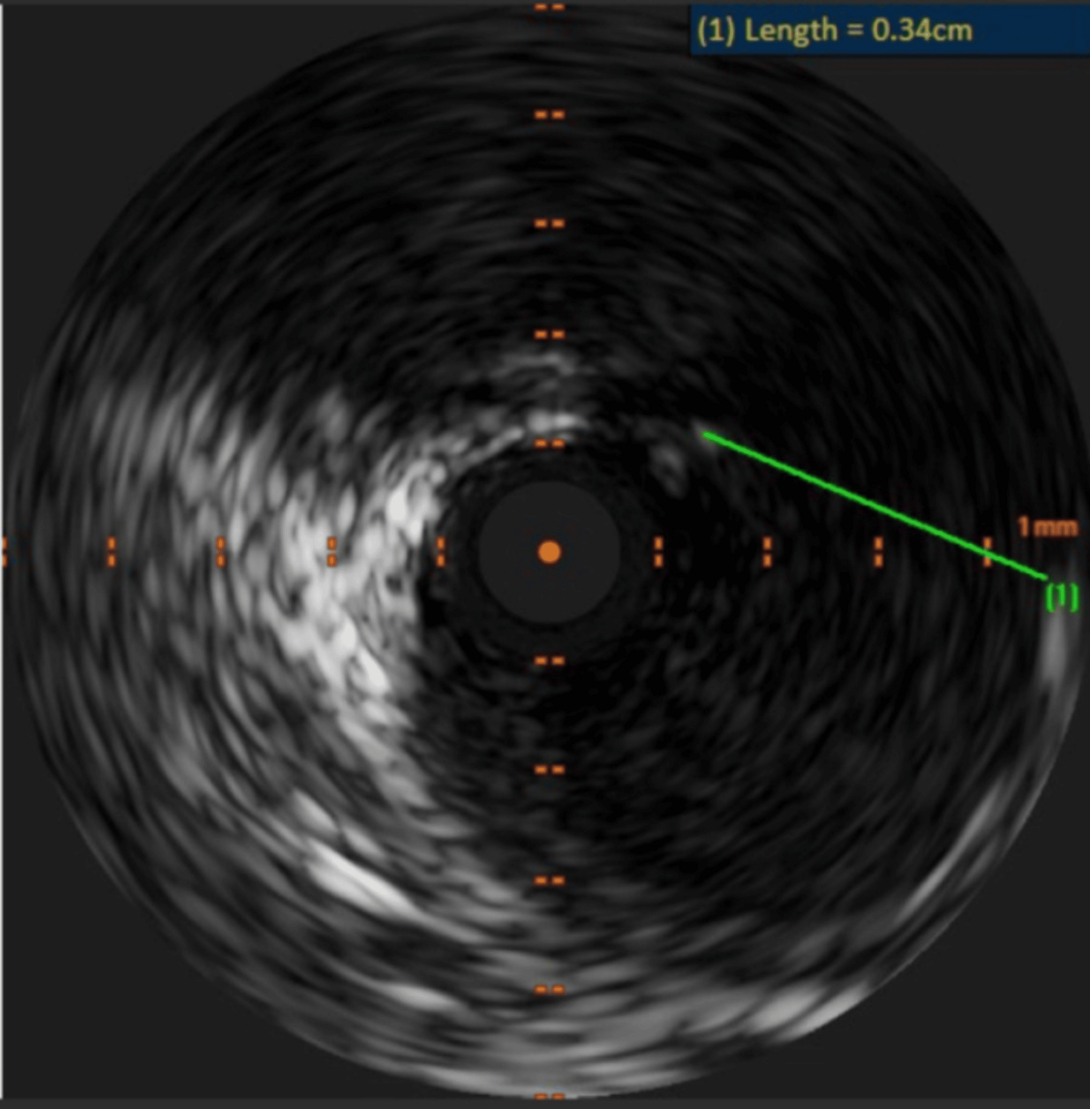
Intravascular ultrasound (IVUS) demonstrating the mouth of the aneurysm

**Figure 4:**
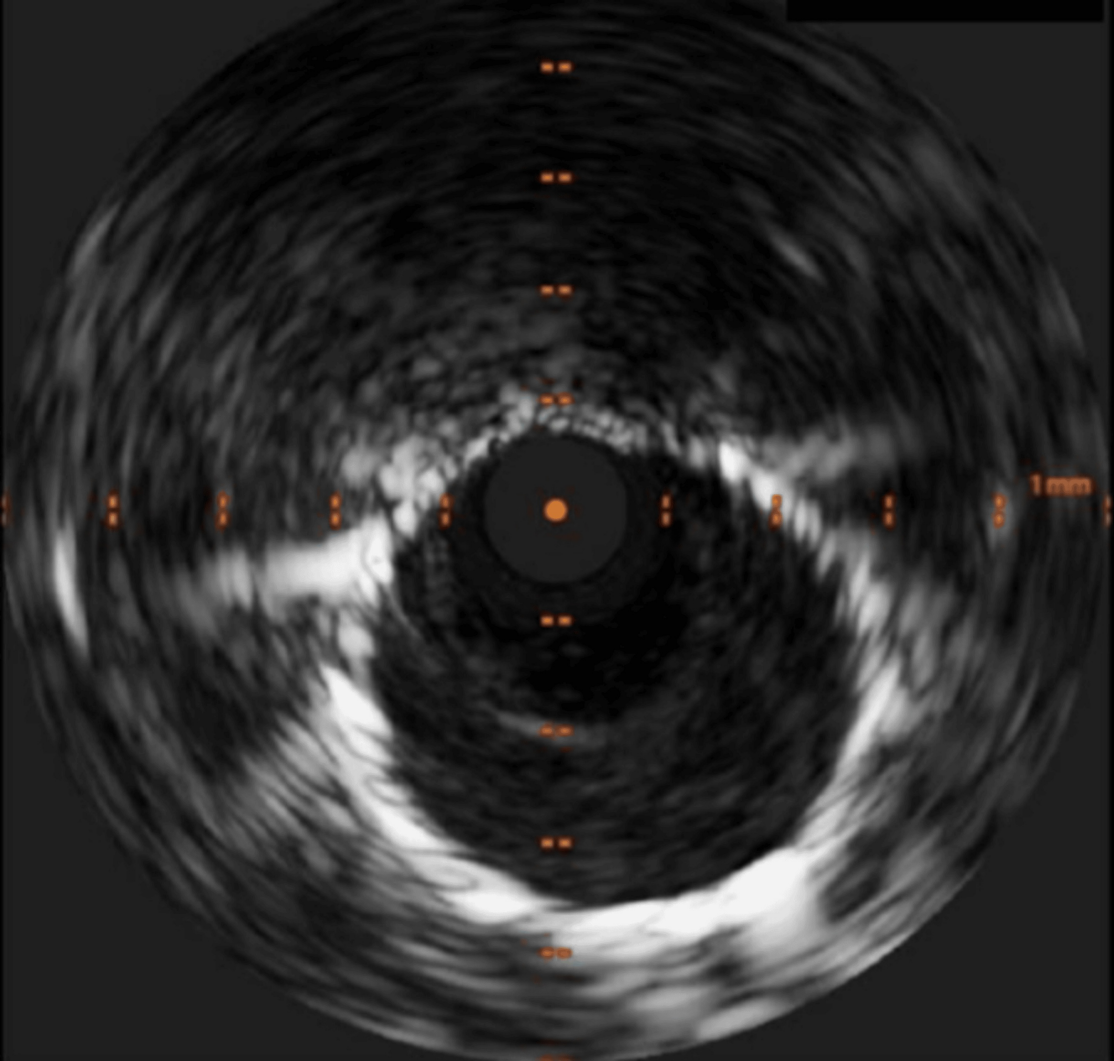
Intravascular ultrasound (IVUS) showing the covered stent

Intravascular ultrasound (IVUS) confirmed a large mouth of the aneurysm as shown in Figure 3, making it unsuitable for coiling. No intimal thickening, thrombus, calcification or mechanical compression was noted on IVUS. Only minor plaque was noted, which is unlikely to cause an acute presentation. After ensuring adequate proximal and distal landing zones on IVUS, we opted to isolate the CAA with a CS (PK PapyrusÒ 5.0x20mm, Biotronik, Germany), which was successful as depicted in Figure 4. A longer drug-eluting stent (BioFreedom™ Ultra 4.5x29mm, Biosensors, Singapore) was then deployed inside the CS, which was post-dilated and optimised with IVUS. Excellent final results were achieved.

The patient was discharged on day 1 post-procedure on DAPT, standard secondary prevention medications, referred for cardiac rehabilitation and follow-up after three months in the clinic was planned, as per our centre protocol.

## Discussion

This case underscores several important considerations in the diagnosis and management of CAA. While atherosclerosis is responsible for most of the adult CAA (around 90%), it is crucial to recognise that nonatherosclerotic causes also exist, such as idiopathic CAA, vasculitis, connective tissue diseases, and post-instrumentation or post-infection sequelae [6]. In this patient, although there are risk factors for atherosclerotic coronary artery disease (CAD), there are no angiographic or IVUS findings suggesting atherosclerotic involvement. Thus, this is considered a nonatherosclerotic CAA - likely idiopathic in nature as there was no history to suggest alternative causes.

Another challenging aspect in managing CAA is the anatomical assessment of the aneurysm and determining its suitability for percutaneous intervention (PCI), if this approach is chosen [7]. In this case, while angiography provided essential information regarding the location and size of the aneurysm, it was insufficient in assessing arterial wall defect size (“mouth” of the aneurysm) and the proximal landing zone for stenting. Therefore, IVUS was performed, which revealed a wide aneurysm mouth, which was unsuitable for coiling due to the high risk of coil embolisation. It also showed an adequate proximal landing zone for the CS placement and facilitated precise stent sizing.

Instrumentation across large aneurysms can present several technical challenges, such as difficulties in passing the guide wire as encountered in this case [8]. Despite attempting various secondary curve sizes and shapes of the guiding wire, there was recurrent looping inside the aneurysm, likely influenced by the turbulent flow in the area. Eventually, wiring through the aneurysmal cavity was adopted, acknowledging that this manoeuvre could potentially lead to aneurysmal wall injury or distal embolisation, particularly if the aneurysmal cavity contained thrombus.

It is probably safer to avoid instrumentation through the aneurysmal cavity. This could potentially be achieved by using guide extensions or trying straight or angulated microcatheters to direct the guide wire away from the aneurysm. However, if this cannot be avoided - whether due to limited resources or time constraints - the operator should remain cautious of potential risks such as distal embolisation or aneurysmal wall rupture [7].

With any coronary interventional procedure, operators should be familiar with the equipment available in their catheterisation lab - particularly CS - before intervening on a CAA. Additionally, interventionists’ experience in managing CAA should be considered.

In this case, the CS was sized one-to-one with the distal vessel reference diameter, as determined by IVUS (5mm). The aneurysmal mouth length was measured by selecting a frame on angiography while the guide wire was being un-looped and remained tent-shaped within the aneurysmal sac prior to becoming completely straight. The distance between the wire's entrance and exit points was then measured (10.6mm) using the angiography system's in-built measuring tool. Following an adequate proximal landing zone as seen on IVUS, a 20mm stent length was selected. This provided almost 5mm in length on each side of the aneurysm. Other methods, such as balloon length measurement with contrast injection or IVUS with co-registration, can also be employed to select stent length [9].

Although commonly used in the percutaneous treatment of CAA, CS are susceptible to target lesion failure (TLF) due to stent thrombosis (ST) and in-stent restenosis (ISR). To address this, an emerging novel trend involving the implantation of overlapping long drug-eluting stents (DES) within the CS, often referred to as the “burying” or - as we prefer to call it - “sandwiching” technique, was implemented in this case [10]. The rationale behind this approach is to promote adequate expansion and optimal apposition of the CS edges while benefiting from the antiproliferative coating of the DES. This strategy aims to facilitate more controlled healing and endothelialisation, thereby reducing the risk of restenosis and improving long-term outcomes [11]. In contrast, using DES alone does not exclude the aneurysm from circulation as done by CS, thus increasing the risk of thrombosis, embolisation and rupture of the aneurysm [12]. Hence, using the sandwich technique is justified.

The antiplatelet strategy for managing CAA in patients who are treated medically, without percutaneous or surgical intervention, remains an area with limited evidence. In some registries, the use of a single antiplatelet agent (either aspirin or clopidogrel) is commonly employed in asymptomatic patients with a low thrombotic risk profile. The addition of a second antiplatelet or an anticoagulant is generally reserved for high-risk patients [13]. There is also some evidence suggesting the potential benefit of vitamin K antagonists in selected Kawasaki disease patients, particularly those with rapidly enlarging aneurysms or those with an absolute internal luminal dimension ≥8mm [14]. However, once the aneurysm is successfully excluded percutaneously with a CS, the choice of antiplatelet therapy becomes less contentious and should be tailored to the individual based on clinical judgement, imaging findings and standard practice for PCI [15,16]. In this case, one year of DAPT followed by long-term aspirin therapy was advised.

## Conclusions

CAA is a rare condition that presents significant diagnostic and therapeutic challenges. Broad-mouthed aneurysms, in particular, require careful procedural planning due to the risk of rupture and distal embolisation. While no specific guidelines exist, medical, percutaneous, and surgical options are available. The “sandwiching” technique - using a drug-eluting stent within a CS - may offer promising long-term outcomes, though further data are needed to validate this approach.
